# “The accuracy of the EOS imaging system to assess hip abnormalities in adolescents and adults:” a systematic review and meta-analysis

**DOI:** 10.1007/s00256-023-04351-2

**Published:** 2023-06-20

**Authors:** Ahmed Alghamdi, Sanjeev Madan, Farag Shuweihdi, Amaka C. Offiah

**Affiliations:** 1 Department of Oncology and Metabolism, University of Sheffield, Damer Street Building, Western Bank, Sheffield, S10 2TH UK; 2 Diagnostic Radiology Department, College of Applied Medical Sciences, King Khalid University, Abha, Kingdom of Saudi Arabia; 3 Sheffield Children’s NHS Foundation Trust, Western Bank, Sheffield, UK; 4 Doncaster &, Bassetlaw Teaching Hospitals, Doncaster, UK; 5 Leeds Institute of Health Sciences, University of Leeds, Leeds, UK

**Keywords:** Systematic review, EOS imaging system, CT scan, Hip, Pelvis

## Abstract

**Objectives:**

To determine the accuracy of the EOS imaging system compared to the gold standard computed tomography (CT) scan, for the measurement of native and postoperative/prosthetic hip parameters in adolescents and adults.

**Methods:**

Medline, Cochrane Systematic Review, and Web of Science databases were searched to obtain relevant articles published between January 1964 and February 2021. All articles published in English. Inclusion and exclusion criteria were developed according to the Population, Intervention, Comparator, Outcome (PICO) framework. Three reviewers independently assessed the quality of included studies using the Quality Assessment of Diagnostic Accuracy Studies (QUADAS-2) checklist. A narrative synthesis of the articles and a meta-analysis were conducted. The heterogeneity exhibited by the effect sizes was obtained using a forest plot, the Q statistic and the I2 index. Reliability coefficients were transformed into Fisher’s Z to normalise their distribution and stabilise the variances. For each meta-analysis, an effect size (average reliability coefficient) and a 95% confidence interval were calculated and presented in a forest plot. The amount of radiation dose between modalities was compared.

**Results:**

The search retrieved 75 articles, six of which met inclusion and exclusion criteria. The meta-analysis included five of these six studies (sample size from 20 to 90). Comparing EOS and CT, the estimated average correlation (effect size) for combined studies was significantly high (r = 0.84, 95% CI = 0.78 to 0.88, *p*-value < 0.001). With respect to Pearson’s correlation between EOS and CT, the estimated average correlation for combined studies was significantly high (r = 0.86, 95% CI = 0.80 to 0.90, *p*-value < 0.001). Average radiation dose for EOS was 0.18 ± 0.05 mGy for the anteroposterior view (AP) and 0.45 ± 0.08 mGy for the lateral view; and for CT was 8.4 to 15.6 mGy.

**Conclusion:**

The EOS imaging system has a high correlation with CT for preoperative and postoperative/prosthetic hip measurements, with considerably lower irradiation of patients.

**Supplementary Information:**

The online version contains supplementary material available at 10.1007/s00256-023-04351-2.

## Introduction

The term femoral version refers to the orientation of the femoral neck in relation to the coronal or transcondylar axis of the distal femur. Femoral anteversion occurs when the femoral neck axis is anteriorly rotated relative to the transcondylar axis and the femoral head axis is anterior to the femur coronal plane, and femoral retroversion occurs when the femoral head-neck axis points are posterior to the femoral coronal plane [1]. The average range of anteversion at birth is from 30 to 40 degrees. These values reduce with growth, remaining in the 10—to—15-degree range for most adults but may be significantly different between populations or contralateral sides [2].

It has been suggested that acetabular orientation is a significant factor when diagnosing and treating hip pathology. Acetabular orientation is defined by two fundamental angles, anteversion and inclination [3], which are regarded as the most critical parameters for determining the quality of the total hip arthroplasty (THA) [4]. Inadequate anteversion and inclination can result in dislocation and femoro-acetabular impingement (FAI) [5]. The elevated inclination may result in a hypo-covered hip component, with the inclination angle determined to be between 40° and 45° [6]. FAI can occur via external hip rotation as a result of a large anteversion, or via internal hip rotation and flexion as a result of a smaller anteversion of approximately 15° to 20°[7].

**Fig. 1:**
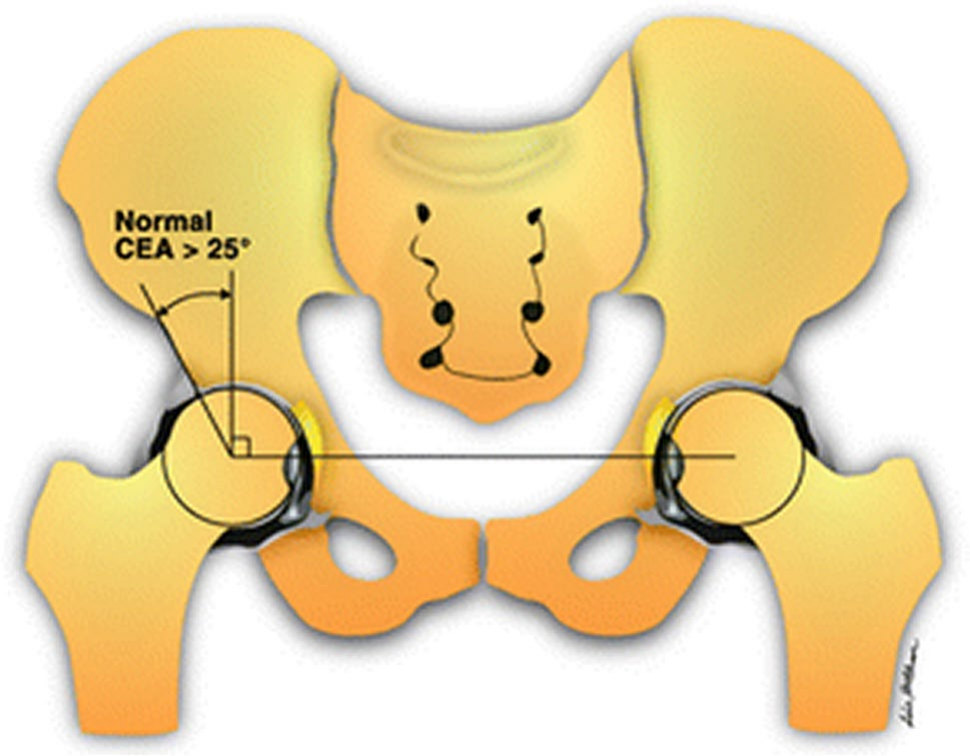
Centre-edge angle (CEA) drawing. The CEA angle is formed between the acetabulum’s perpendicular and lateral margin [11]. Reproduced with permission of the American Roentgen Ray Society from ‘Imaging Evaluation of Developmental Hip Dysplasia in the Young Adults,’ authored by Beltran et al., published in the Review journal, 200, 5, 1077-1088. Copyright© [2013] American Roentgen Ray Society

It is well known that the acetabular dysplasia results in hip pain that leads to the dysfunction of the hip that could later cause hip osteoarthritis [8, 9]. Acetabular lateral centre edge angles (LCEA) are important in acetabular dysplasia measurements [10]. It is called lateral centre edge angle (LCEA) and centre edge angle (CEA), interchangeably. The angle is formed by a vertical line crossing the centre of the femoral head and a marginal line to the acetabulum’s lateral margin (Fig. 1) [11]. Periacetabular osteotomy tends to normalise these angles to 25° to 40° [12]. Lateral centre edge angles less than 20° are considered dysplastic [13]. Similarly in prosthetic hips optimal acetabular cup inclination of 30° to 50° is considered optimal [14, 15].

Acetabular anteversion of 10° to 20° is considered normal [16]. In prosthetic hips surgeons aim to achieve acetabular cup anteversion of 5° to 25° [14]. Achieving these normal angles in periacetabular osteotomy and in THA is important for stability of the hip and survival of the native and prosthetic hip.

Inability to recognise abnormal hip anteversion or retroversion early in life may have a detrimental effect on the hip’s range of motion and stability [17, 18]. For hip preservation surgery, peri-acetabular osteotomy is the most promising technique for treating dysplasia in adolescents and young adults [19]. Whether pre- or postoperatively, the use of appropriate radiological imaging modalities is critical for guiding orthopaedic interventions [17, 18].

According to some studies, conventional radiography is incapable of measuring femoral version accurately and should be avoided in favour of more precise methods such as computed tomography (CT) [20, 21]. Computed tomography is currently the reference method for measuring femoral version [20]. However, its clinical utility is constrained by issues such as excessive radiation exposure, which can have a detrimental effect on patients, particularly children [22]. Magnetic resonance imaging (MRI) is a potential alternative for determining femoral version; however, it is costly, time-consuming, and prone to motion artefacts. When anaesthesia is required for the examination, the associated costs and risks of MRI increase [23]. The EOS imaging system may offer an alternative to the techniques mentioned previously. It requires a lower radiation dose than CT, and the sterEOS software enables the creation of 3-dimensional (3D) images [24].

**Fig. 2:**
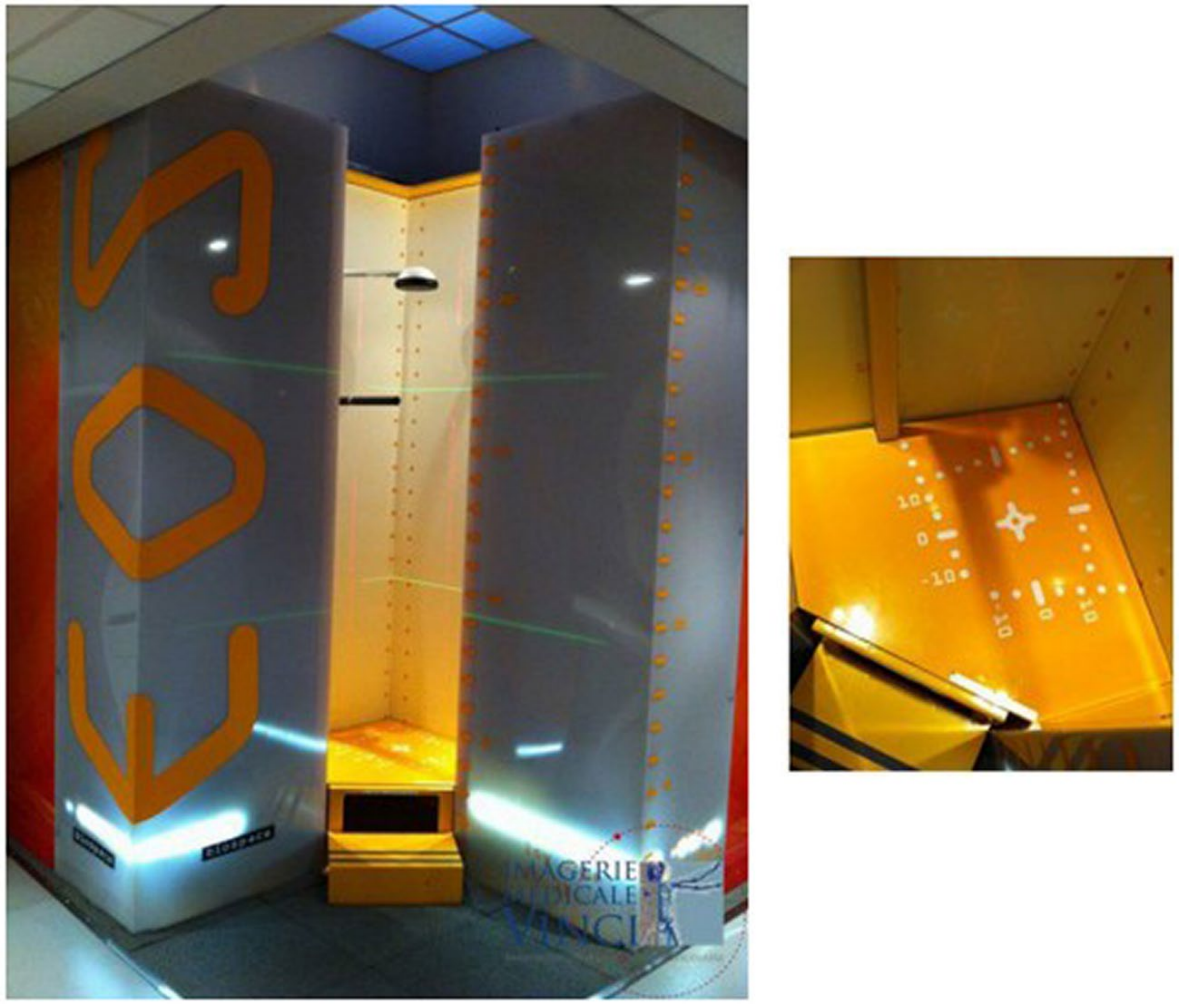
EOS imaging system 2D/3D (left) and its marked ground (right) [27]. Reproduced from ‘Musculoskeletal Imaging in Progress: The EOS Imaging System,’ authored by Marc Wybier and Philippe Bossard, published in 2012, Volume 80, Issue 3, pages 238-243. Copyright © 2012 Société franc¸aise de rhumatologie. Published by Elsevier Masson SAS. All rights reserved

**Fig. 3:**
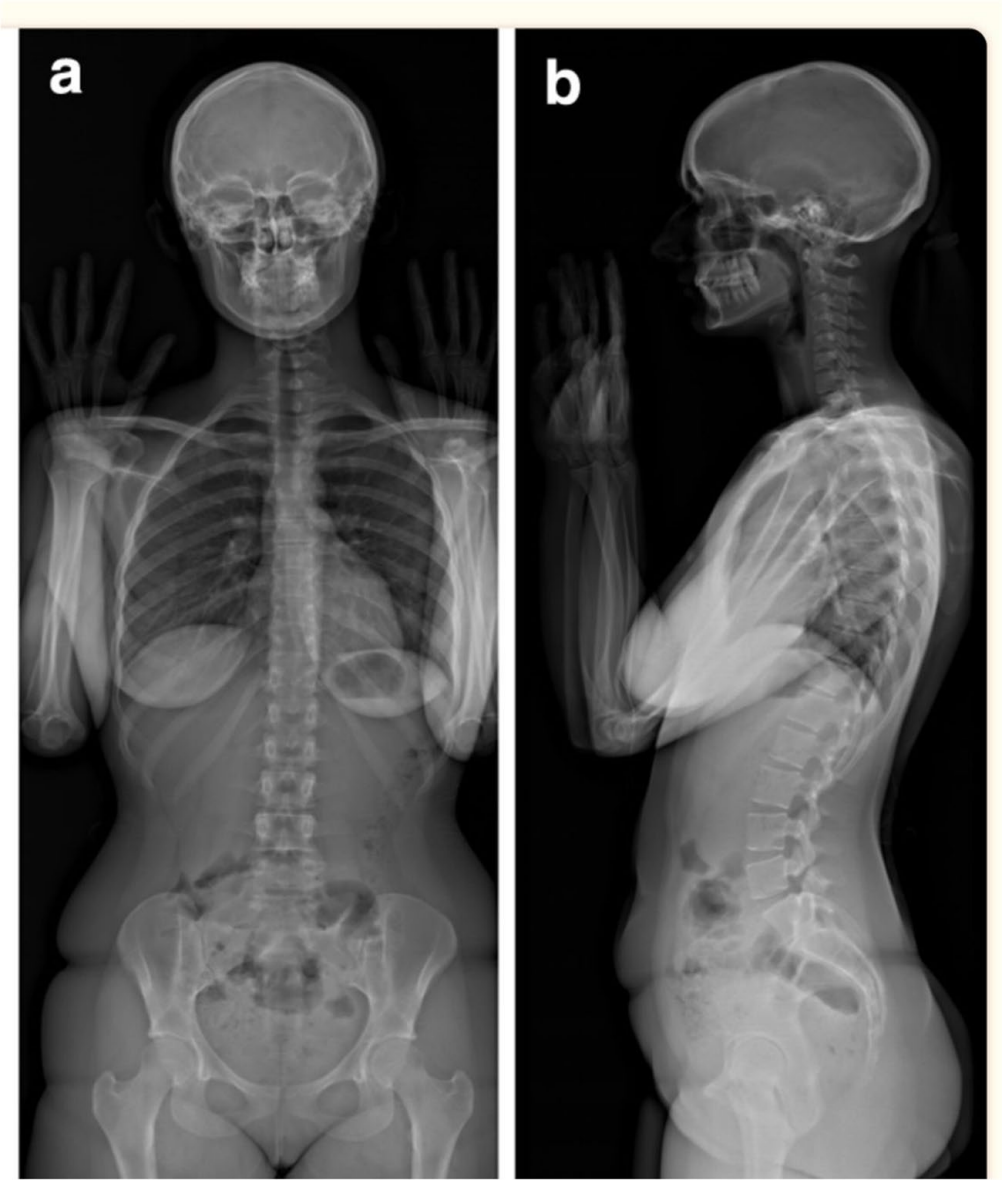
Full body on simultaneously captured images of anteroposterior (AP) (left) and lateral (LAT) (right) [28]

**Fig. 4:**
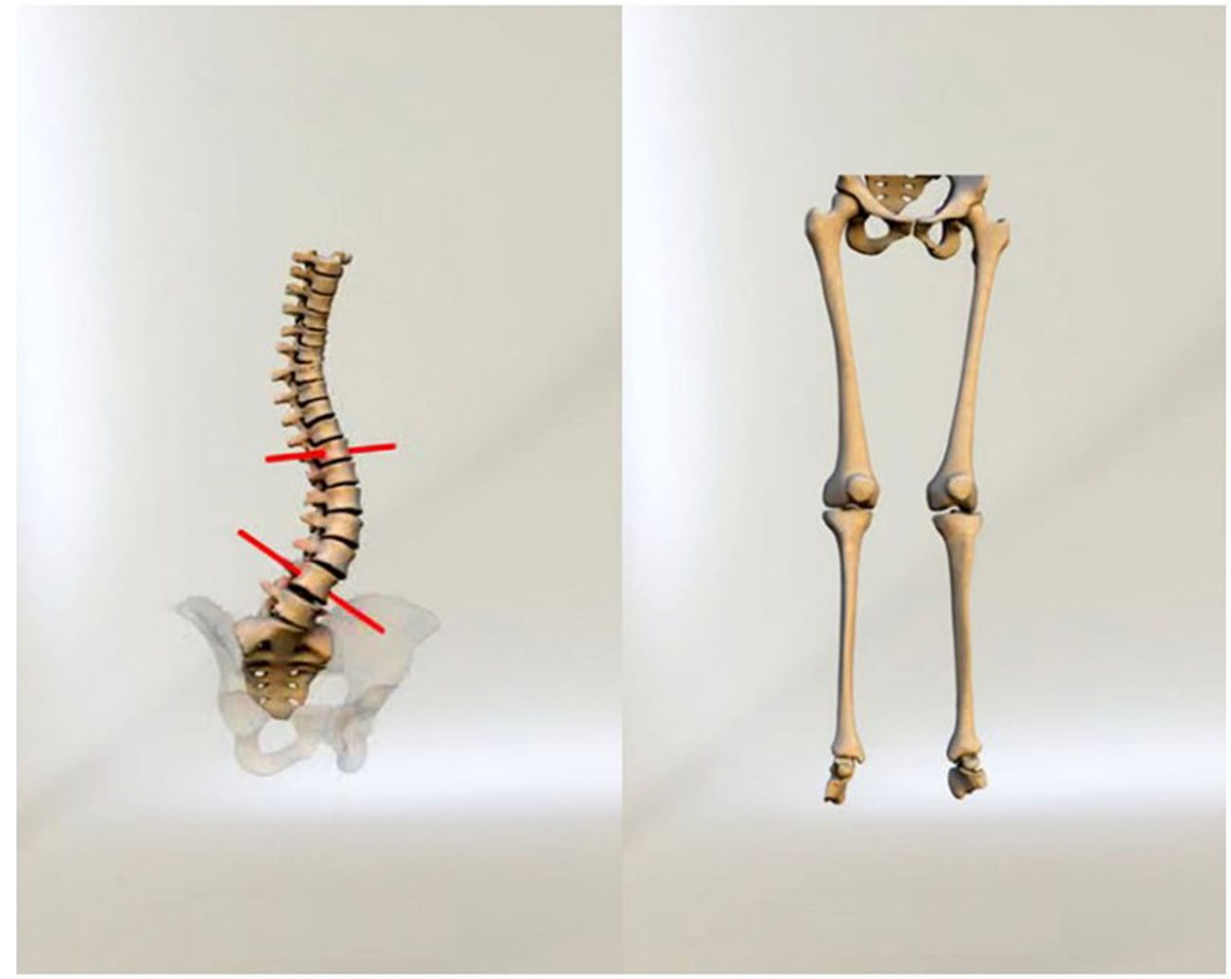
Reconstruction of 3D model from 2D EOS imaging of pelvis with spine (left) and with lower extremity (right)[29]

A new X-ray imaging device has been developed which incorporates this novel detection technology. It consists of two co-located pairs of 45-cm wide linear radiation sources and detectors that are perpendicular to one another and positioned both frontally and laterally [25, 26] (Fig. 2) [27]. Biplanar X-ray images are captured simultaneously within this X-ray imaging device, dubbed EOS 2D/3D, via the vertical movement of two pairs of X-ray tubes and detectors. This vertical movement covers a 170 cm high × 45 cm wide area, producing high-quality, high-contrast anteroposterior (AP) and lateral (LAT) X-ray images in as little as 10–25 s [25, 26] (Fig. 3) [28]. Additionally, rigorous 3D reconstructions of the pelvis, vertebrae and other skeletal sites can be produced (Fig. 4) [29].

In this study, the literature on the accuracy of the EOS system for hip assessment compared to the reference method of CT has been systematically reviewed. The objectives of this study are as follows:

- To determine the accuracy of the EOS imaging system compared to CT for measuring native hip/pelvic parameters.
- To determine the accuracy of the EOS imaging system compared to CT for measuring postoperative/prosthetic hip parameters.

## Methods

This systematic review is registered on PROSPERO (registration number: CRD42021234026). The study was conducted according to the Preferred Reporting Items for Systematic reviews and Meta-Analysis (PRISMA) guidelines [30].

### Search strategy

Pre-defined search terms (EOS, EOS imaging system, EOS system, biplanar low dose radiography, low-dose biplanar imaging system, EOS imaging technology, EOS X-ray, EOS – imaging, low-dose biplanar radiographs, EOS stereoradiography, 3D stereoradiography, CT scan, CT, computed tomography, hip, pelvis and acetabulum) were used to search the following databases: Medline, Cochrane Systematic Review database, and Web of Science (Supplementary information). The search included all articles published in English between January 1864 and February 2021. To ensure the inclusion of any relevant papers not retrieved by the initial search, the reference lists of the included papers were also reviewed.

### Study selection

Inclusion and exclusion criteria were developed according to the following (PICO) framework:

Population: adolescents and adults undergoing pre- or postoperative hip or pelvic imaging; Intervention or index test (IT): EOS imaging system; Comparator: Computed tomography (CT) scan; Outcome(s): Accuracy of the EOS imaging system compared to CT for measuring native hip/pelvic parameters; accuracy of the EOS imaging system compared to CT for measuring postoperative/prosthetic hip parameters.

#### Inclusion criteria

Studies involving humans; studies of adolescents and adults with hip abnormalities; use of the EOS imaging system to measure hip/pelvic parameters and comparison of EOS system measurements to those obtained using CT as the gold standard method.

#### Exclusion criteria

Studies conducted on phantoms or animals; manuscripts or abstracts in languages other than English and abstracts of conference meetings, case reports and review articles as it could not be peer reviewed.

After inserting the articles retrieved into a reference manager (Endnote), one reviewer (AMA) exported their details to MS Excel spreadsheets, removed duplicated articles and performed abstract and title screening. To ensure the eligibility of all included studies, the reviewer also carried out full-text screening.

### Data extraction

**Fig. 5:**
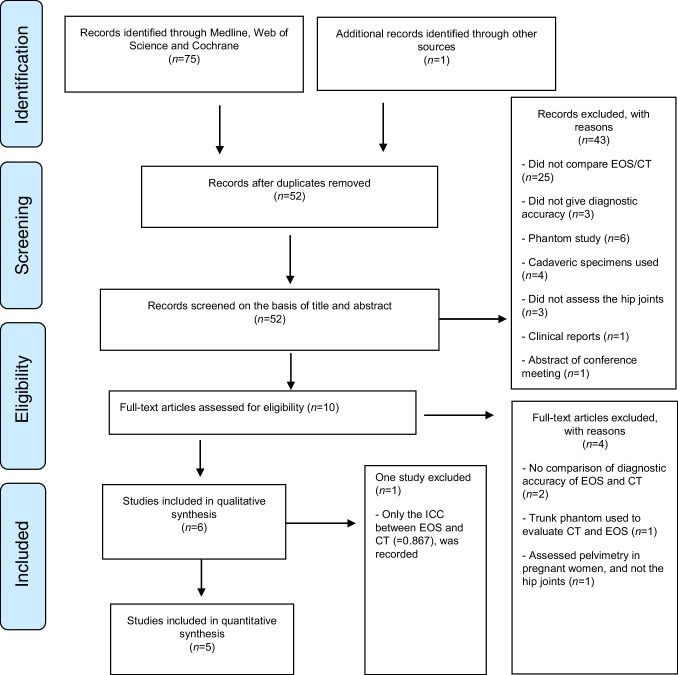
PRISMA flow chart for search results and study selection

The studies were retrieved and evaluated as shown in the PRISMA flow diagram [30] in Fig. 5. A standardised data extraction form was used to collect the following information from included studies: first author, study design, year of publication, reference test (EOS), CT scan protocol, sample size and main findings.

### Quality assessment

Quality assessment was performed by three reviewers (AMA, SM, ACO) using the Quality Assessment of Diagnostic Accuracy Studies (QUADAS-2) checklist [31]. This instrument contributes to the assessment of eligibility and risk of bias of studies included in a review. The checklist includes four key domains: patient selection; index test; reference standard; and flow and timing. The reviewers independently scored each study, rating the items as having a low, high or unclear risk of bias.

### Strategy for data synthesis

A narrative synthesis of the findings was performed for the final included studies. This includes a summary of the study characteristics and outcomes related to the precision of the EOS imaging system compared to CT scans in the pre- and postoperative assessment of hip abnormalities in adolescents and young adults.

Statistical meta-analyses were conducted by assuming a random-effects model, and each type of reliability (correlation and intraclass correlation coefficients (ICC)) was weighted by the inverse variance [32]. Meta-analyses were conducted using a random-effects model to investigate simple correlation and ICC.

All the extracted coefficients were transformed into Fisher’s Z to normalise their distribution and stabilise the variances [33]. For each meta-analysis, an effect size (average reliability coefficient) and a 95% confidence interval were calculated [34] and presented in a forest plot. Subgroup analysis was conducted to assess the difference between non-operative pelvis measurements vs. post-operative hip measurements. Subgroup analysis was only applied to simple correlation data since no subgroups were obtained for the other underlying measurements.

The heterogeneity exhibited by the effect sizes was obtained using a forest plot, tau ( between-study deviation), Q statistic and the I^2^ index [35]. Based on the rule of thumb [36], the I2 values 0 to 40% indicates ‘may not be important’, 30% to 60% indicates ‘may represent moderate heterogeneity’, 50% to 90% indicates ‘may represent substantial heterogeneity’ and 75% to 100% indicate ‘considerable heterogeneity’.

To assess for publication bias, a funnel plot was used to determine the relationship between reliability coefficients (effect sizes) and the corresponding estimated standard errors [37]. Sensitivity analysis was implemented using eight visual representations to detect outlying studies influencing the results of the meta-analysis [38].

The R statistical software version 4.2.2 (R foundation for statistical computing, Vienna, Austria) was used for all statistical analyses; the “meta” and “metafor” package were used for the meta-analysis [39].

## Results

### Search strategy

The initial search yielded 75 articles. Twenty-three articles were excluded after duplicates and 43 were excluded after title and abstract screening for some reasons as they did not compare EOS/CT (*n* = 25), they did not give diagnostic accuracy (*n* = 3), they were phantom studies (*n* = 6), they used cadaveric specimens (*n* = 4), they did not assess the hip joints (*n* = 3), they were clinical reports (*n* = 1) and they were abstracts of conference meeting (*n* = 1). The remaining nine articles were selected for full-text evaluation. One additional article was included based on the reference lists of included articles, this article was not found in the search process and added manually from a personal reference list. The full-text screening of these ten articles resulted in the exclusion of four studies. Two were excluded for not comparing the diagnostic accuracy of EOS and CT. One study was excluded for using a trunk phantom to evaluate the CT and EOS method, and one for performing a pelvimetry and evaluating pelvic dimensions rather than the hip joints. Six eligible papers were therefore included in the qualitative synthesis (Fig. 5). For quantitative synthesis, one study was excluded due to insufficient reporting of data (only an ICC between EOS and CT of 0.867, was reported).

### Study characteristics

The six articles included were published between 2012 and 2021. There were 222 participants in total (range from 20 to 90). Table 1 provides the following characteristics of all included studies: author, year of publication, sample size, sample age, reference test (CT), EOS imaging protocol and main findings.

**Table 1:** Characteristics of eligible studies

Study	Author (year)	Study design and recruitment date	Sample size (*n*)	Mean age (years)	Reference test (CT scan)	EOS imaging protocol	Main findings
1	Buck, F. M., et al. (2012)	Not reported	35 patients (12 male)	Women: 64 range 46–89 Men: 67 range 57–78	40- MDCT scanner (Philips) axial images (140 kV; 300mAs; matrix 512 × 512; pitch .426; .45 mm reconstruction increment; 1 mm reconstruction thickness)	40 kV and 250 mAs	High correlation for all measurements with *P* < 0.001 High correlation with ICC of 0.952 (95% CI, 0.905–0.976) and 0.938 (95% CI, 0.878–0.969) with CT, for femoral torsion and tibial torsion respectively High correlation with ICC of 0.943 (95% CI, 0.886–0.971) and 0.959 (95% CI, 0.918–0.979) with EOS, for femoral torsion and tibial torsion respectively High correlation for femoral anteversion (AV) angle with IC = 0.952
2	Folinais, D., et al. (2013)	Retrospective study (between November 2009 and March 2011	43 lower limbs/ 30 patients (15 male)	53.2 ± 20.4	Helicat CT scanner/ 40-slice	Not reported	Mean difference of 6.3° and 6.8° between EOS and CT, respectively; for femoral torsion, with *P* = 0.6 Mean difference of 3.9° for tibial torsion High correlation with ICC with EOS 0.93 and 0.86 for femoral and tibial torsion, respectively; ICC with CT was 0.90 and 0.92 for femoral and tibial torsion, respectively For anteroposterior in EOS, radiation dose was 0.18 ± 0.05 mGy, and 0.45 ± 0.08 mGy for lateral position For CT, radiation dose was 8.4 to 15.6 mGy High correlation with ICC approximately 0.9 for both EOS and CT High correlation between EOS and CT with r = 0.93 for femoral anteversion (AV) angle
3	Tokunaga, K., et al. (2018)	Not reported	90 patients (15 male)	60	80-slice CT machine (Aquilion PRIME, Toshiba)	Not reported	High correlation between EOS and CT for implant angle measurements of THA, with correlation coefficients (rho) as follows: stem antetorsion = 0.8861, cup anatomical anteversion = 0.763 and cup radiographic inclination = 0.679, with *P* < 0.0001 High correlation between EOS and CT in measurement values, but a difference of 5° in cup anteversion and stem antetorsion due to outliers
4	Fritz, Benjamin et al. (2019)	Retrospective study	50 patients (29 male)	69.7 range 53–87	64-slice CT scanner (Philips) 120 kV/250 mAs/collimation (64 × 0.625 mm)/ rotation time (0.5 s.)/1 mm slice thickness for axial images)	AP image (83–95 kV and 200-280 mA)/ For lateral image (102-120 kV and 200-320 mA)	High correlation between EOS and CT with ICC ≥ 0.8 for 2D and 3D acetabular coverage For global 3-D acetabular coverage measurements, difference between CT and BPR of only 0.9% with standard deviations of 3.6% and 3.0%, respectively
5	Esposito, Christina I., et al. (2020)	Prospective study	20 patients	Not reported	Not reported	Not reported	Mean difference values between EOS and CT: 4° ± 4° for femoral anteversion, 3° ± 2° for acetabular anteversion and 2° ± 2° for acetabular inclination With CT, inter-rater correlation greater than 0.78 for all hip angle measurements; slight difference with EOS may be due to outliers High correlation with EOS as follows: For acetabular inclination (Cronbach’s α = 0.83) For acetabular anteversion (Cronbach’s α = 0.89) For femoral components (Cronbach’s α = 0.89)
6	Mayr, Hermann O., et al. (2021)	Observational study	34 femora measured/ 19 patients (4 male)	45.5 ± 19.8	Not reported	Not reported	Both hip measurements (15 patients) One side measurement (four patients) Eleven hips had no torsional malalignment; fourteen had reduced anteversion (less than ten degrees) or retroversion (less than zero degrees) Nine hips had increased anteversion (more than 20 degrees) High correlation between for the assessment of femoral anteversion angle (AV), r = 0.855 in patients with regular AV (*n* = 34) Moderate correlation between (r = 0.495) in patients with reduced AV (less than 10 degrees) involving retroversion (less than zero degrees), (*n* = 14) Low correlation r = 0.292, in patients with increased AV (more than 20 degrees), (*n* = 9) Significant correlation in all measurements (*n* = 34; *P* < 0.001), in physiological AV (*n* = 11; *P* = 0.001), in decreased AV and retroversion (*n* = 14; *P* = 0.072) and in increased AV (*n* = 9; *P* = 0.446) High agreement on AV measurements between all 3 examiners with ICC = 0.911 using EOS and ICC = 0.934 using CT Good inter-observer reliability with Cronbach’s α values of 0.955, 0.934 for EOS and CT, respectively No correlation observed in patients with torsional malalignment

*AP* anteroposterior, *AV* anteversion, *CT* computed tomography, *ICC* intraclass correlation coefficient, *MDCT* multi-detector computed tomography, *THA* total hip arthroplasty

### Quality assessment

The results of quality assessment of the six included studies are shown in Table 2. Most studies had a low risk of bias in all domains. However, two studies had an unclear risk of bias in the flow and timing domain, since the interval between CT and EOS was not stated [40, 41].

**Table 2:** Quality assessment of included studies

Study	Risk of bias (1 = low, 2 = medium, 3 = high)				Applicability concerns			
	Patient selection	Index test	Reference standard	Flow and timing	Patient selection	Index test	Reference standard	Flow and timing
Buck, F. M., et al. (2012)	1	1	1	1	1	1	1	1
Folinais, D., et al. (2013)	1	1	1	2	1	1	1	1
Tokunaga, K., et al. (2018)	1	1	1	1	1	1	1	1
Fritz, Benjamin et al. (2019)	1	1	1	1	1	1	1	1
Esposito, Christina I., et al. (2020)	1	1	1	1	1	1	1	1
Mayr, Hermann O., et al. (2021)	1	1	1	2	1	1	1	1

### Diagnostic accuracy of EOS in measuring hip/pelvic parameters compared to CT

Six studies compared the diagnostic accuracy of EOS for hip/pelvic parameter measurements to that of CT. Three of these studies evaluated the diagnostic accuracy of femoral and tibial torsion measurements. Folinais et al. [40] discovered a high correlation between EOS and CT, with r = 0.93 and 0.89, respectively, when measuring femoral and tibial torsion. Mayr et al. [41] found a high correlation between EOS and CT in measuring anteversion angle (AV) in patients with suspected torsional malalignment, with r = 0.855. Buck et al. [42] reported that the ICC for femoral torsion CT measurements was 0.952 (95% CI, 0.905–0.976) and for tibial torsion CT measurements was 0.938 (95% CI, 0.878–0.969). The ICC for femoral EOS measurements was 0.943 (95% CI, 0.886–0.971) and for tibial EOS measurements was 0.959 (95% CI, 0.918–0.979).

Additionally, two studies compared the diagnostic accuracy of EOS and CT for hip/pelvic parameter measurements in patients undergoing THA [43, 44]. Tokunaga et al. [43] concluded that there was a high correlation between EOS and CT measurements, with correlation coefficients (rho) of 0.6973 (*P* = 0.0001) for cup radiographic inclination, 0.763 (*P* = 0.0001) for cup anatomical anteversion, and 0.8861 (*P* = 0.0001) for stem antetorsion. Esposito et al. [44] discovered a high correlation between CT and EOS measurements: r = 0.87 (*P* = 0.01) and r = 0.89 (*P* = 0.01) for cup inclination, r = 0.85 (*P* = 0.01) and r = 0.78 (*P* = 0.01) for cup anteversion, and r = 0.91 (*P* = 0.01) and r = 0.88 (*P* = 0.01) for femoral anteversion.

Moreover, Fritz et al. [45] documented a high correlation between EOS and CT for measuring acetabular coverage with ICC = 0.867.

### Pooled correlation between EOS and CT for hip/pelvic parameter measurements

**Fig. 6:**
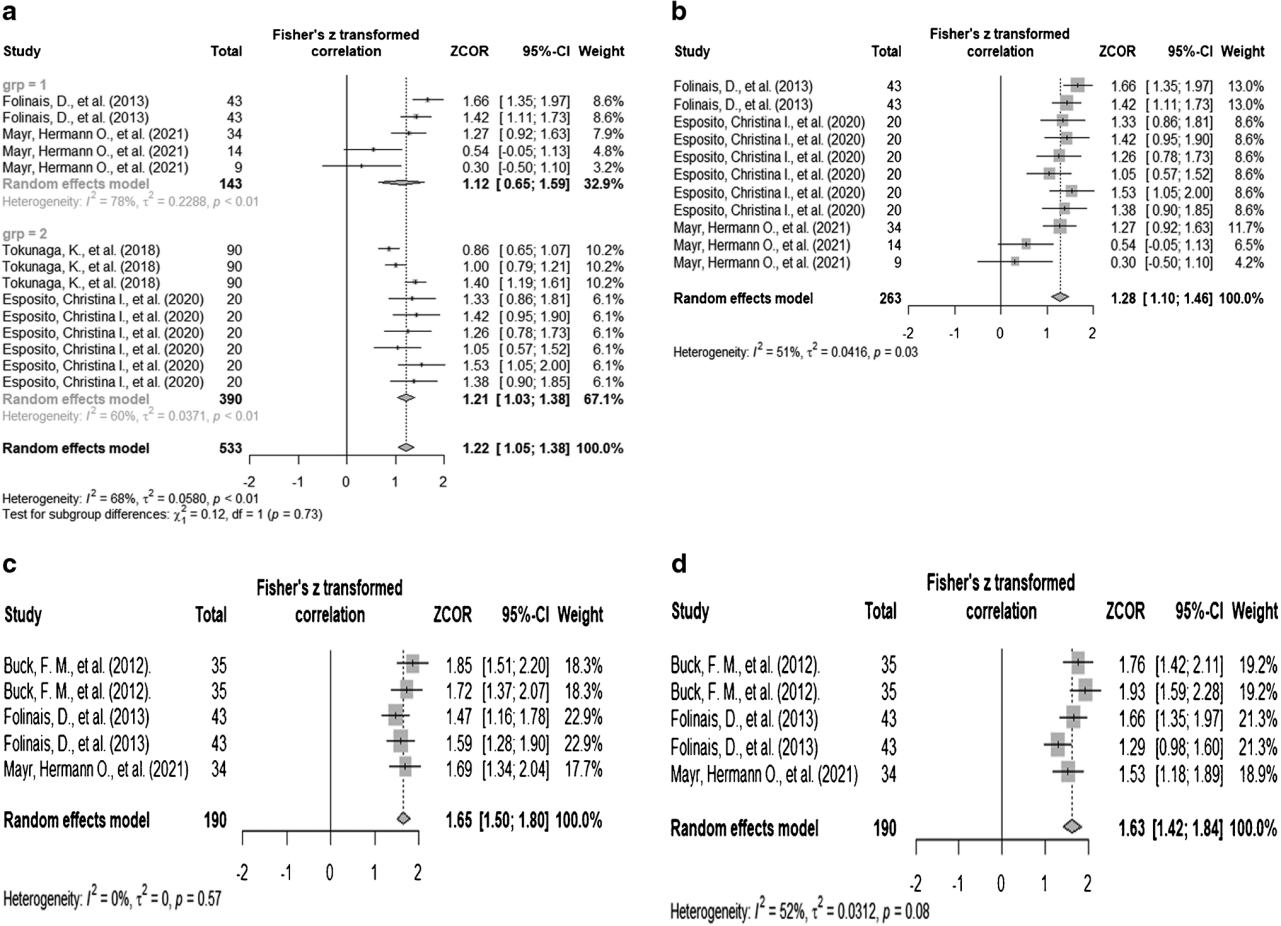
**a**–**d** Forest plots. **a**, **b** showing correlation between EOS and CT using Fisher’s Z-transformation, and subgroup analysis (grp 1 = non-operative pelvis measurements, grp 2 = post-operative hip measurements) (**a**). Fisher’s transformed Pearson’s correlation coefficient (**b**). **c** Forest plot for Fisher’s transformed ICC coefficient for CT.** d** Forest plot for Fisher’s transformed ICC coefficient for EOS

We grouped five studies (*n* = 222) that correlated the diagnostic accuracy of EOS with hip/pelvic parameter measurements compared to CT. For the correlation between the EOS and CT, the estimated average correlation (effect size) for combined studies was significantly high (*r* = 0.84, 95% CI = 0.78 to 0.88, *p*-value < 0.001) with moderate heterogeneity (*I*^*2*^ = 68%; Fig. 6a) and *tau* = 0.24. The subgroup analysis showed the effect size for non-operative pelvis measurements (r = 0.81, 95% CI: 0.57 to 0.92, tau = 0.48 *I*^*2*^ = 78%) was similar and post-operative hip measurements (r = 0.84, 95% CI: 0.77 to 0.88, *tau* = 0.19 I^*2*^ = 60%), and the test showed that there was no significant difference (chi-squared = 0.12, *p*-value = 0.73) (Fig. 6a).

With respect to Pearson’s correlation between the EOS and CT, the estimated average correlation for combined studies was significantly high (*r* = 0.86, 95% CI = 0.80 to 0.90, *p*-value < 0.001) with moderate heterogeneity (*I*^*2*^ = 51%; Fig. 6b) and *tau* = 0.20. Regarding the ICC for CT, the effect size was significantly high (ICC = 0.929, 95% CI = 0.905 to 0.947, *p*-value < 0.001) with no heterogeneity (*I*^*2*^ = 0.0%; Fig. 6c) and *tau* = 0.20. Regarding the ICC for EOS, the effect size was significantly high (ICC = 0.93, 95% CI = 0.89 to 0.95, *p*-value < 0.001) with moderate heterogeneity (*I*^*2*^ = 52; Fig. 6d) and *tau* = 0.18.

**Fig. 7:**
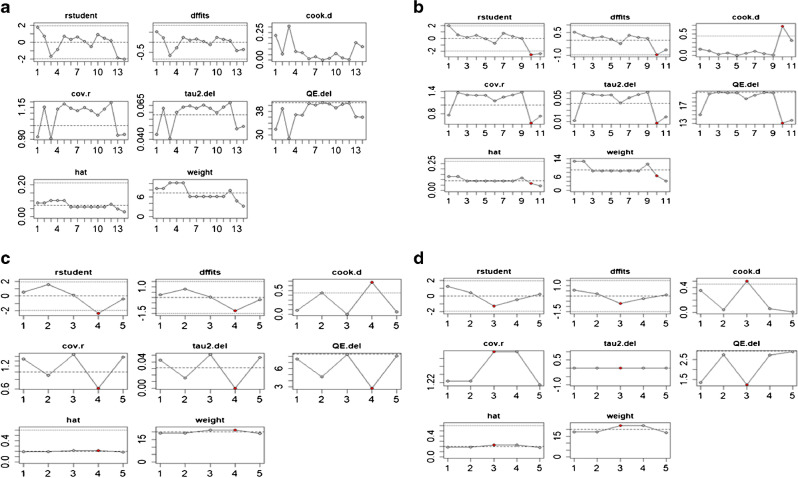
**a**–**d** Sensitivity analysis for outliers using eight methods of visualization for simple correlation coefficient between EOS and CT (**a**), Pearson’s correlation coefficient between EOS and CT (**b**), ICC coefficient for CT (**c**) and ICC coefficient for EOS (**d**)

The sensitivity analyses are shown in Fig. 7. A sensitivity analysis of outliers for simple correlation coefficient between EOS and CT detected no influential study. The sensitivity analysis for Pearson’s correlation coefficient between EOS and CT detected no influential study. Sensitivity analysis of outliers indicated no influential study in the ICC coefficient for CT. Sensitivity analysis of outliers indicated no influential study in the ICC coefficient for EOS.

**Fig. 8:**
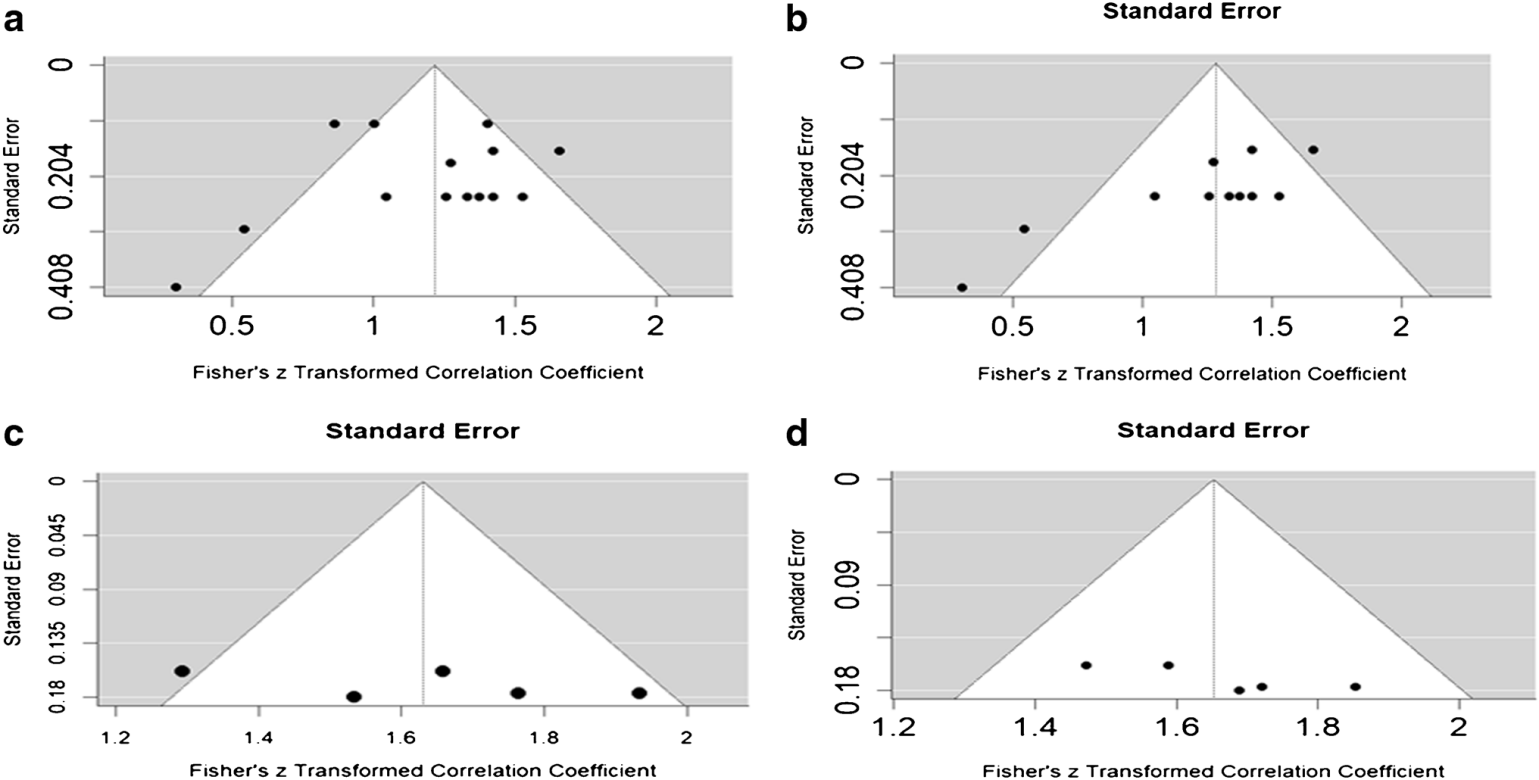
**a**–**d** Funnel plots for Fisher’s transformed simple correlation coefficient between EOS and CT (**a**), Fisher’s transformed Pearson’s correlation coefficient between EOS and CT (**b**), Fisher’s transformed ICC coefficient for CT (**c**) and Fisher’s transformed ICC coefficient for EOS (**d**)

The funnel plots (Fig. 8) were mostly symmetrical. Using Rosenthal’s method, the failsafe number was 26,403 (*p*-value < 0.001), which exceeds Rosenthal’s rule of thumb (5* k* + 10 = 80, where *k* (number of effect size in meta-analysis) = 14), indicating that publication bias in our meta-analysis is minimal. The funnel plot was mostly symmetrical. Using Rosenthal’s method, the failsafe number was 14,448 (*p*-value < 0.001), which exceeds Rosenthal’s rule of thumb (5* k* + 10 = 65, where *k* (number of effect size in meta-analysis) = 11), indicating that publication bias in our meta-analysis is minimal. Regarding publication bias, the funnel plot and the failsafe number, which is 18,262 (*p*-value < 0.001) and exceeds Rosenthal’s rule of thumb, indicate that publication bias in our meta-analysis is minimal. Regarding publication bias, the funnel plot and the failsafe number, which is 18,220 (*p*-value < 0.001) and exceeds Rosenthal’s rule of thumb, indicate that publication bias in our meta-analysis is minimal.

## Discussion

This systematic review and meta-analysis demonstrate the EOS system's diagnostic accuracy in measuring hip/pelvic parameters in comparison to CT. Overall, high correlation has been reported between these imaging modalities.

While high correlation was suggested between the EOS system and the reference method CT scan to measure femoral and tibial torsion [40, 42], no correlation was observed between EOS and CT in the assessment of the femoral AV angle in patients with torsional malalignment [41]. This may be explained by the fact that patients with torsional malalignment are unable to keep their legs in the neutral position during the CT scan, which limits the reliability and accuracy of this method [46]. This issue could be overcome by the use of EOS imaging [41]. On the other hand, the EOS system cannot be used in patients who are unable to stand or hold the correct position during examination [40, 41]. In such patients, the accuracy of EOS images might be influenced by motion artifact.

A significant correlation was identified between EOS and CT when measuring hip angles (femoral anteversion and acetabular anteversion angle) in patients who had undergone THA [43, 44]. This suggests that the EOS system may provide reliable hip measurements even after THA. However, large differences in acetabular anteversion between EOS and CT [44] were found in one patient, and similar differences in femoral anteversion were found in four patients. This finding may be explained by difficulties in determining anatomic landmarks by less experienced radiologists. Therefore, more training is needed to allow for the use of EOS as an alternative to measuring the position of hip components after THA.

High correlation between EOS and CT has been reported in measuring 2D and 3D acetabular coverages [45]. However, this study was conducted on patients having no hip abnormalities. In contrast, anterior and posterior coverage difference is supposed to be higher between these imaging modalities in patients with hip abnormalities such as symptomatic femoroacetabular impingement (FAI), as increasing in anterior pelvic tilt were discovered [47, 48]

The advantage of an EOS system compared to CT is lower irradiation of the patients [49, 50]. This is significant as the radiation dose should always be as low as possible and always within the “As Low As Reasonably Achievable” (ALARA) principle. It has been reported that the EOS system emits a radiation dose that is 8–10 and 800–1000 times lower than that of conventional radiography and CT scans, respectively [51, 53].

Notably, average radiation dose for EOS was 0.18 ± 0.05 mGy for the anteroposterior view (AP) and 0.45 ± 0.08 mGy for the lateral view, and for CT was 8.4 to 15.6 mGy [40]. This indicates that EOS would be a beneficial alternative for patients needing vast orthopaedic examinations including many imaging radiographs over a period of time.

Finally, a number of important limitations need to be considered. First, this review only included studies in English, which may have led to the exclusion of significant data presented in studies published in other languages. Secondly, since all studies reviewed were conducted within a specific age group (adolescents and adults), our findings may have missed important data from studies involving children.

This is the first systematic review to compare the accuracy of the EOS imaging system to that of CT scan, considered the gold standard for hip assessment. The present review demonstrates that the EOS system can be an alternative to CT for measuring and evaluating hip/pelvic parameters, with the advantage of lower irradiation.

The current study shows that the EOS system has been validated for femoral anteversion by comparison with CT measurements, but not for the native acetabulum. Therefore, further study is needed to devise a method of measuring acetabular anteversion using EOS images and validated against CT measurements of native hips.

### Supplementary Information

Below is the link to the electronic supplementary material.

Supplementary file1 (DOCX 18 KB)

## Data Availability

The data that support the findings of this study are available from the corresponding author, [A.M.A], upon reasonable request.

## Declarations

### Statistics and biometry

Farag Shuweihdi is a research fellow and a Medical Statistician at the Leeds Institute of Health Sciences, University of Leeds, Leeds.

### Methodology

• Systematic review.

• Meta-analysis.
