# Supplementary material for: “The accuracy of the EOS imaging system to assess hip abnormalities in adolescents and adults:” a systematic review and meta-analysis
Source: Skeletal Radiol. 2023 Jun 20;53(1):29–42. doi: 10.1007/s00256-023-04351-2 (PMC10661804; doi:10.1007/s00256-023-04351-2)
Supplement: Supplementary file 1 — Supplementary file1 (DOCX 18 KB) [file 256_2023_4351_MOESM1_ESM.docx]

**Supplementary information**

A- Search strategy

A.1. Medline

| # | Search | Result |
| --- | --- | --- |
| 1 | exp Hip/ or hip.mp. | 167163 |
| 2 | exp Pelvis/ or pelvis.mp. | 70963 |
| 3 | acetabulum.mp. or exp Acetabulum/ | 14733 |
| 4 | 1 or 2 or 3 | 234646 |
| 5 | EOS imaging system.mp. | 52 |
| 6 | EOS imaging technology.mp. | 2 |
| 7 | EOS system.mp. | 66 |
| 8 | EOS X-ray.mp. | 11 |
| 9 | EOS.mp. | 4468 |
| 10 | EOS - Imaging.mp. | 141 |
| 11 | biplanar low dose radiography.mp. | 2 |
| 12 | low-dose biplanar imaging system.mp | 3 |
| 13 | low-dose biplanar radiographs.mp. | 11 |
| 14 | EOS stereoradiography.mp. | 5 |
| 15 | EOS stereoradiography system.mp. | 3 |
| 16 | 3D stereoradiography.mp. | 2 |
| 17 | 5 or 6 or 7 or 8 or 9 or 10 or 11 or 12 or 13 or 14 or 15 or 16 | 4476 |
| 18 | exp Tomography, X-Ray Computed/ or CT scan.mp. | 473406 |
| 19 | CT.mp. | 361409 |
| 20 | computed tomography.mp. | 287951 |
| 21 | 18 or 19 or 20 | 709719 |
| 22 | 17 and 21 | 132 |
| 23 | 4 and 22 | 26 |
| 24 | limit 23 to English language | 23 |

A.2 Web of science

| **#** | **Searches** | **Results** |
| --- | --- | --- |
| 1 | TS=(hip) | 323,012 |
| 2 | TS=(pelvis) | 129,727 |
| 3 | TS=(acetabulum) | 22,375 |
| 4 | #1 OR #2 OR #3 | 436,712 |
| 5 | TS=(EOS imaging system) | 1,502 |
| 6 | TS=(EOS imaging technology) | 224 |
| 7 | TS=(EOS system) | 8,870 |
| 8 | TS=(EOS X-ray) | 4,301 |
| 9 | TS=(EOS) | 28,634 |
| 10 | TS=(EOS - Imaging) | 2,744 |
| 11 | TS=(biplanar low dose radiography) | 88 |
| 12 | TS=(low-dose biplanar imaging system) | 78 |
| 13 | TS=(low-dose biplanar radiographs) | 69 |
| 14 | TS=(EOS stereoradiography) | 44 |
| 15 | TS=(EOS stereoradiography system) | 33 |
| 16 | TS=(3D stereoradiography) | 88 |
| 17 | #5 OR #6 OR #7 OR #8 OR #9 OR #10 OR #11 OR #12 OR #13 OR #14 OR #15 OR #16 | 28,754 |
| 18 | TS=(CT scan) | 222,291 |
| 19 | TS=(CT) | 725,397 |
| 20 | TS=(computed tomography) | 894,322 |
| 21 | #18 OR #19 OR #20 | 1,239,613 |
| 22 | #17 AND #21 | 337 |
| 23 | #4 AND #22 | 55 |
| 24 | #23 Refined by: LANGUAGES: (ENGLISH) | 52 |

A.3 Cochrane

| **#** | **Searches** | **Results** |
| --- | --- | --- |
| 1 | (acetabular anteversion OR femoral anteversion):ti,ab,kw | 107 |
| 2 | (EOS):ti,ab,kw | 1120 |
| 3 | (biplanar low dose radiography):ti,ab,kw | 2 |
| 4 | #2 OR #3 | 1121 |
| 5 | (CT):ti,ab,kw | 77481 |
| 6 | (computed tomography):ti,ab,kw | 19679 |
| 7 | #5 OR #6 | 87606 |
| 8 | #4 AND #7 | 68 |
| 9 | #1 AND #8 | 0 |
